# Biomarkers of endothelial dysfunction in relation to impaired carbohydrate metabolism following pregnancy with gestational diabetes mellitus

**DOI:** 10.1186/s12933-014-0138-3

**Published:** 2014-10-04

**Authors:** Christian S Göbl, Latife Bozkurt, Rajashri Yarragudi, Thomas Prikoszovich, Andrea Tura, Giovanni Pacini, Renate Koppensteiner, Alexandra Kautzky-Willer

**Affiliations:** Department of Gynecology and Obstetrics, Division of Feto-Maternal Medicine, Medical University of Vienna, Vienna, Austria; Department of Internal Medicine III, Division of Endocrinology and Metabolism, Unit of Gender Medicine, Medical University of Vienna, Waehringer Guertel 18-20, Vienna, A-1090 Austria; Metabolic Unit, Institute of Biomedical Engineering, National Research Council, Padova, Italy; Department of Internal Medicine II, Division of Angiology, Medical University of Vienna, Vienna, Austria

**Keywords:** Endothelial dysfunction, Insulin resistance, Gestational diabetes mellitus

## Abstract

**Background:**

History of gestational diabetes mellitus (GDM) identifies a very young population of females predisposed for type 2 diabetes and cardiovascular disease. Endothelial dysfunction might represent a shared precursor of both disorders. Hence, this study aimed to characterize endothelial biomarkers in relation to impaired insulin sensitivity and progression to overt diabetes early after index pregnancy.

**Methods:**

108 women with previous GDM and 40 controls were included three to six months after delivery and underwent specific metabolic assessments including a frequently sampled intravenous glucose tolerance test and an oral glucose tolerance test. Diabetes progression was assessed in females with pGDM over 10 years of follow-up. Circulating sICAM-1 (intracellular-adhesion-molecule-1), sVCAM-1 (vascular-cell-adhesion-molecule-1) and sE-selectin, representing biomarkers of endothelial dysfunction were assessed at baseline and annually over five years.

**Results:**

Endothelial biomarkers were significantly associated with insulin sensitivity (sICAM-1: r = -0.23, p = 0.009; sVCAM-1: r = -0.22, p = 0.011; sE-selectin: r = -0.21, p = 0.018) as well as with GDM status and parameters of subtle inflammation. Analysis of long-term trajectories revealed constantly elevated sICAM-1 (p = 0.033) and sE-selectin (p = 0.007) in 25 subjects with diabetes progression. Accordingly, sE-selectin levels at the early post partum visit predicted a later development of the disease (HR =1.02 95%CI 1.01 to 1.04, p = 0.013), however, this was attenuated after adjustment for BMI.

**Conclusions:**

Elevated circulating markers of endothelial dysfunction in young females with GDM history might reflect an early stage on the pathway to the manifestation of future cardiometabolic disorders. Timely identification of women at high risk and optimization of follow-up management might provide an opportunity to prevent disease progression.

## Background

Endothelial dysfunction characterizes a state of dysbalance between vasoconstriction and vasodilatation predisposing atherosclerosis and cardiovascular disorders (CVD), the principal complications of type 2 diabetes [1,2]. This process starts already several years before the clinical manifestation of overt diabetes as also prediabetic hyperglycemia is accompanied by inflammation and endothelial damage [1-4]. In addition it was supposed, that dysfunctional endothelium by itself aggravates impaired glucose disposal, mainly due to the loss of vasoactive properties mediated by insulin (i.e. total blood flow as well as the regulation of capillary perfusion in insulin sensitive tissues) [3,5,6]. At the same time the expression of cellular adhesion molecules (CAMs) and selectins on the surface of the endothelium is up-regulated, inducing tethering and firm adhesion of leucocytes, maintaining inflammation and hence further promoting an atherogenic milieu [6,7]. Consequently, endothelial dysfunction was suggested to be an “unifying factor” for both: cardiovascular and hyperglycemic disorders [8]. However, clinical consequences in different risk populations are not thoroughly investigated until now [5].

Females with history of gestational diabetes mellitus (GDM) are of particular interest for studying the relationship between endothelial dysfunction, impaired insulin sensitivity and cardiometabolic disorders, as this high risk entity represents the earliest stage of a prediabetic condition suffering from an increased risk for developing type 2 diabetes [9] but also CVD [10,11]. In this context, previous observational studies indicated that obesity and impaired glucose metabolism during the pregnant state is associated with markers of endothelial dysfunction [12], which might also be apparent in the postpartal period [13,14]. However, data on this issue is contradictory [15].

Therefore, this report aims to assess three main objectives: (i) to describe the association of soluble cellular adhesion molecules including, sICAM-1 (soluble intracellular adhesion molecule-1), sVCAM-1 (soluble vascular cell adhesion molecule-1) as well as soluble E-selectin (sE-selectin) with impaired insulin sensitivity early after pregnancy with GDM; (ii) to examine long-term trajectories of these parameters as well as (iii) their association with the later development of type 2 diabetes. As a secondary objective, the impact of impaired insulin sensitivity and history of GDM with increased carotid intima media thickness (IMT) a well established marker of early atherosclerosis should be examined in a subgroup.

## Methods

### Study participants

The study design of the “Vienna Post-Gestational Diabetes Project” was reported elsewhere e.g. [16-18]. For the present report we included data of 108 females 3–6 months after pregnancy with GDM (pGDM) and 40 controls (women after gestation without GDM) with available data of soluble adhesion molecules or IMT (2 pGDMs and 1 control subject were excluded from the original study cohort due to missing data of these parameters). Diagnoses of GDM was performed according to [19] by a 75 g-OGTT between June 1999 and December 2003.

### Experimental procedures and calculations

Participants received a metabolic characterization at the baseline examination including soluble biomarkers of endothelial dysfunction (i.e. sICAM-1, sVCAM-1, sE-selectin) as well as a 75 g-OGTT after at least 8 hours fasting with measurements of glucose, insulin and C-peptide. A 3 h frequently sampled intravenous glucose tolerance test (FSIGT) was additionally performed at the baseline visit (details of this examination are reported elsewhere e.g. [17,20]) to assess the insulin sensitivity index (SI_FSIGT_) by minimal model analysis according to [21,22]. For group based comparisons pGDMs were divided into insulin sensitive (pGDM-IS) or insulin resistant subjects (pGDM-IR) by using the previously defined cut-off of SI = 2.8 × 10^−4^ min^−1^/(μU/ml) [23]. Insulin secretion from FSIGT (ΔAIRG) was estimated by the incremental short-term insulin response (3–10 min) after intravenous glucose load [24]. Insulin sensitivity was additionally estimated from OGTT data by using the oral glucose insulin sensitivity index (OGIS) [25] and insulin secretion from OGTT data was assessed by the insulinogenic index (IGI = Δinsulin30/Δglucose30) [26].

Biomarkers of endothelial dysfuction were reexamined within 12 to 18 month after the baseline visit in females with pGDM over a period of approximately five years (median: 59 month, IQR: 50 - 62 month). OGTTs were annually repeated in the pGDM subgroup over up to 10 years of follow-up (until March 2013) to identify subjects with diabetes manifestation (median: 81.5 month, IQR: 51 – 142 month for subjects included in the present report). Overt diabetes was diagnosed, if FPG or 2 h post load glucose levels exceeded 126 mg/dl or 200 mg/dl.

IMT measurements were performed by a ATL (Advanced Technology Laboratories, Bothell, Washington 98021 USA), HDI 5000 system. A linear array, 12–5 MHz Probe was employed. To keep variabilities to a minimum the primary measurements were performed in the distal common carotid artery, 1 cm proximal to the carotid bulb on the far vessel wall in two angles of the transducer; secondary measurements were performed proximally and distally to this point in the common and in the proximal internal carotid artery with assessment of the far vessel wall by an experienced coinvestigator blinded to any additional clinical information. The average of these measurements was used for further analyses. Carotid IMT were performed in n =69 subjects (n =54 pGDMs and n =15 controls) 60 month (IQR: 19.5 – 65 month) after the baseline examination. 14 subjects had a repeated measurement during this period.

The study was approved by the Ethics Committee of the Medical University of Vienna and performed in accordance with the Declaration of Helsinki. All participants gave written informed consent.

### Laboratory methods

Plasma glucose (Beckman, Fullerton, CA); Insulin (radioimmunoassay, Serono Diagnostics); C-Peptide (radioimmunoassay, CIS Bio International); Adhesion molecules (ELISA, British Biotechnology Product Ltd.); PAI-1 (ELISA, Technoclone); IL-6 (ELISA, R&D Systems); usCRP (N High Sensitivity CRP Reagent, BN Systems); sICAM-1, sVCAM-1, sE-selectin (ELISA, British Biotechnology Product Ltd.).

### Statistical analysis

Baseline data were summarized by means and standard deviations (SD) in case of continuous variables as well as by counts and percentages in case of categorical variables. Differences of metric scaled parameters in more than two groups were assessed by analysis of variance (ANOVA). Linear trend analysis was used to assess trends of endothelial biomarkers according to subgroups categorized by their status of insulin sensitivity. The influence of continuous covariates on sICAM-1, sVCAM-1 and sE-selectin was assessed by correlation analysis (Pearson’s product moment correlation or Spearman’s rank correlation) and multivariable regression models, respectively. Fractional polynomials (by using a backward selection algorithm for suitable transformations as proposed by [27] with a family-wise error rate of 5%) were used to model the relationship between endothelial biomarkers and insulin sensitivity. Data transformations were performed in case of skewed distributed baseline variables: PAI-1, triglycerides: ln(x); usCRP, IL-6: ln(x +1). The proportional hazard model (and a survival regression for sensitivity analyis to account for interval censoring) was used to examine the associations of sICAM-1, sVCAM-1 and sE-selectin with the incidence of type 2 diabetes during the follow-up period. Analyses of longitudinal data were examined by linear mixed effects models (random intercepts and random slopes by subjects). For some models the random slope was excluded if this was necessary to achieve convergence. A spatial exponential covariance structure was used to model the correlations between repeated measurements. sICAM-1 and sVCAM-1 were log transformed (ln(x)) and sE-selectin was square root transformed (sqrt(x)) for longitudinal analyses. IMT data (83 measurements in 69 subjects) was analysed by using a random intercept model accounting for correlated residuals due to repeated measurements in some subjects. In case of linear relationships regression coefficients (β) represent the mean change in the dependent variable for the increase of the independent variable by one unit. If the independent variable is a factor (e.g. GDM status) they can be interpreted as the mean differences of factor levels (i.e. GDM vs. NGT), adjusted for other variables in the model.

Statistical analysis was performed with R (V3.0.2) and contributed packages (particularly the R-packages “mfp”, “nlme”, “survival” for data analysis as well as “lattice” and “beeswarm” for visualizations) [28]. The two sided significance level was set to 0.05. No adjustment for multiplicity was considered for this observational study.

## Results

### Associations of endothelial biomarkers with insulin sensitivity, GDM status and subclinical inflammation at the early postpartum period

**Figure 1:**
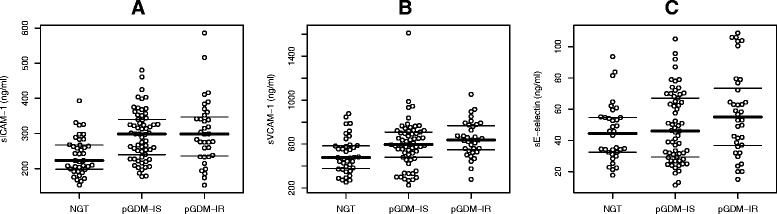
**Bee swarm plot of endothelial biomarkers at baseline in different subgroups: normal glucose tolerant controls (NGT) as well as females after pregnancy with GDM grouped by insulin resistance status (pGDM-IS: insulin sensitive; pGDM-IR: insulin resistant): A: sICAM-1; B: sVCAM-1; C: sE-selectin.** Lines indicate first, second (median) and third quartiles.

**Figure 2:**
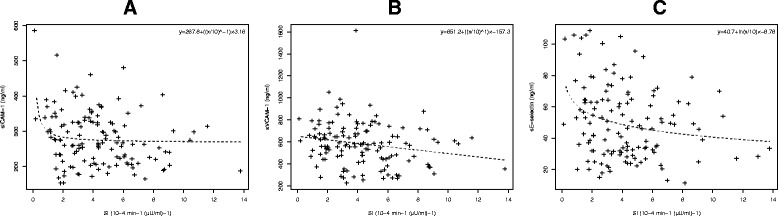
**Association of baseline sICAM-1 (A), sVCAM-1 (B) and sE-selectin (C) with SI levels and the regression curve derived from regression analysis based on fractional polynomials.**

A brief summary of the study sample in Table 1 shows that biomarkers of endothelial dysfunction at the baseline examination were highest in the pGDM-IR subgroup (Figure 1). This observation was also confirmed by analyses of continuous data, as sICAM-1, sVCAM-1, and sE-selectin were inversely related to SI_FSIGT_ 3–6 month after delivery (Table 2). Regression models based on fractional polynomials revealed monotonic but non-linear relationships between insulin sensitivity at baseline (SI_FSIGT_) and sICAM-1 as well as sE-selectin, which were used in multivariable models (Figure 2). However, no association were observed between endothelial biomarkers and insulin secretion (Table 2).

**Table 1:** **Baseline characteristics categorized by insulin sensitivity status**

	**n**	**Controls**	**n**	**pGDM-IS**	**n**	**pGDM-IR**	**p-value**
Age (years)	40	31.2 ± 5.7	63	32.8 ± 4.2	39	32.9 ± 5.7	0.203
BMI (kg/m2)	37	25.2 ± 5.6	63	25.0 ± 3.5	39	30.5 ± 5.6	<0.001
OGIS, ml min^−1^ m^−2^	39	490.1 ± 71.0	63	463.6 ± 68.6	39	395.3 ± 72.7	<0.001
SI, 10^−4^ min^−1^ (μU/ml)^−1^	38	5.4 ± 2.6	63	5.3 ± 2.3	39	1.7 ± 0.6	<0.001
ΔAIRg (μu/ml)	38	54.4 ± 42.8	63	36.0 ± 22.7	39	50.3 ± 38.6	0.017
sICAM-1 (ng/ml)	39	238.0 ± 54.1	61	295.3 ± 70.5	35	304.1 ± 92.2	<0.001*
sVCAM-1 (ng/ml)	39	502.3 ± 164	61	600.3 ± 220	35	647.7 ± 159	0.001*
sE-selectin (ng/ml)	34	45.1 ± 18.5	61	48.6 ± 22.4	36	57.4 ± 27.4	0.026*

Data are means ± standard deviation. BMI (body mass index), OGIS (oral glucose insulin sensitivityindex), SI (insulin sensitivity index), ΔAIRg (acute insulin response), sICAM-1 (soluble intracellular adhesion molecule-1), sVCAM-1 (soluble vascular cell adhesion molecule-1), sE-selectin (soluble E-selectin).

*p-values are based on a test of linear trend.

**Table 2:** **Correlation of endothelial biomarkers and characteristics of the study population**

		**sICAM-1**			**sVCAM-1**			**sE-selectin**	
	**n**	**r**	**p-value**	**n**	**r**	**p-value**	**n**	**r**	**p-value**
age	140	0.05	n.s.	140	0.04	n.s.	136	0.09	n.s.
BMI	136	0.25	**0.004**	136	0.21	**0.016**	132	0.35	**<0.001**
RRS	131	0.14	n.s.	131	0.10	n.s.	128	0.12	n.s.
RRD	131	0.11	n.s.	131	0.18	**0.036**	128	0.13	n.s.
SI (FSIGT)*	133	−0.23	**0.009**	133	−0.22	**0.011**	129	−0.21	**0.018**
OGIS (OGTT)*	137	−0.18	**0.033**	137	−0.23	**0.007**	133	−0.12	n.s.
ΔAIRg (FSIGT)*	133	−0.09	n.s.	133	0.04	n.s.	129	−0.04	n.s.
IGI (OGTT)*	139	−0.12	n.s.	139	0.03	n.s.	135	−0.10	n.s.
Total-Cholesterol	140	−0.03	n.s.	140	0.10	n.s.	136	−0.02	n.s.
LDL-Cholesterol	137	0.08	n.s.	137	0.13	n.s.	133	0.02	n.s.
HDL-Cholesterol	140	−0.30	**0.001**	140	0.06	n.s.	136	−0.16	n.s.
ln(Triglycerides)	140	0.12	n.s.	140	−0.02	n.s.	136	0.12	n.s.
ln(usCRP)	116	0.28	**0.003**	116	0.18	n.s.	114	0.04	n.s.
ln(IL-6)	93	0.13	n.s.	93	0.16	n.s.	94	0.24	**0.019**
ln(PAI-1)	132	0.29	**<0.001**	132	0.08	n.s.	128	0.21	**0.016**
Fibrinogen	140	0.25	**0.003**	140	0.04	n.s.	136	0.25	**0.003**

Data are number of observarion (n) and correlation coefficients (r). BMI (body mass index), RRS (systolic blood pressure), RRD (diastolic blood pressure), SI (insulin sensitivity index derived from the FSIGT), OGIS (oral glucose tolerance index derived from the OGTT), ΔAIRG (acute insulin response to glucose derived from the FSIGT), IGI (insulinogenic index), HDL (high density lipiprotein), LDL (low density lipoprotein), usCRP (ultrasensitive C-reactive protein), IL-6 (interleukin-6), PAI-1 (plasminogen activator inhibitor 1).

*Spearman’s rank correlation as used instead of Pearson’s product moment correlation due to nonlinear monotonic associations or outliers.

Multivariable analyses including status of GDM during pregnancy in addition to age, BMI and SI_FSIGT_ showed that females with history of GDM had significantly elevated levels of adhesion molecules as compared to control subjects: sICAM-1: β =55.2, 95%CI 27.5 to 82.9, p <0.001; sVCAM-1: β =109.2, 95%CI 31.6 to 186.9, p =0.006. However, no differences were observed for sE-selectin (β =4.30, 95%CI −5.25 to 13.85, p =0.374 for pGDM vs. controls), which was best explained by BMI levels (β =1.39, 95%CI 0.43 to 2.35, p =0.005). The models explained 21.2% (sICAM-1), 8.3% (sVCAM-1) and 11.2% (sE-selectin) of the variances, respectively.

Correlation analyses showed significant associations between serum lipid levels as well as parameters of subclinical inflammation and endothelial biomarkers (mainly sICAM-1 and sE-selectin, Table 2). sICAM-1 remained significantly associated with HDL-cholesterol and usCRP also after adjustment for age, BMI, SI_FSIGT_ and status of GDM (GDM vs. controls): HDL-cholesterol: β = −0.97, 95%CI −1.86 to −0.09, p = 0.032; usCRP: β = 62.9, 95%CI 9.80 to 116.1, p = 0.021 (for log transformed data), whereas associations of inflammatory parameters and sE-selectin were attenuated.

We found no evidence of strong effect modification (i.e. interaction) of continuous associations by GDM status.

### Association of endothelial biomarkers with the later development of overt diabetes

**Figure 3:**
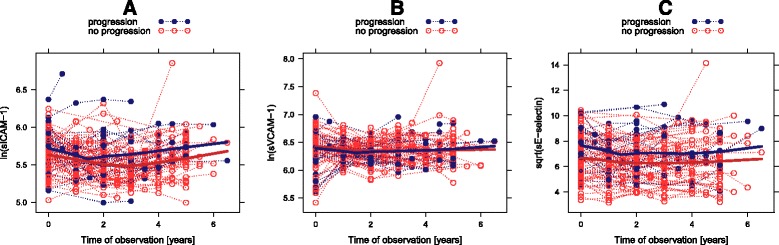
**Trajectories of soluble endothelial biomarkers in subjects after pregnancy with GDM categorized for diabetes progression (blue) or no progression (red) over the study period: sICAM-1 (A), sVCAM-1 (B), sE-selectin (C).**

Trajectories of endothelial biomarkers are visualized in Figure 3 and showed no major time related changes during the follow-up examinations of females following pregnancies with GDM. However, longitudinal analysis revealed constantly increased sICAM-1 (β = 0.108, 95%CI 0.009 to 0.207, p = 0.033 (for ln transformed data)) and moreover elevated sE-selectin levels (β =0.96, 95%CI 0.27 to 1.64, p = 0.007 (for sqrt transformed data)) in n =25 female subjects with progression to overt diabetes. The results remained constant, if subjects with a follow-up period of less than four years (and no diabetes progression) were excluded. No interactions between trajectories of endothelial biomarkers and progression status were observed. In accordance, sE-selectin levels at the early postpatum visit was significantly related to diabetes progression (HR =1.02 95%CI 1.01 to 1.04, p = 0.013 (per ng/ml)) in the pGDM subgroup. This association remained constant after adjustment for age (p = 0.024) but attenuated after additionally adjusting for BMI (p = 0.273). Regarding adhesion molecules baseline sVCAM-1 levels appeared to be not related with incident type 2 diabetes (0.474), whereas a tendency was observed for sICAM-1 (HR = 1.01 95%CI 1.00 to 1.01, p = 0.050 (per ng/ml)). We performed an additional sensitivity analysis treating diabetes progression as interval censored time to event data, however, the basic conclusions remained constant.

### Association of intima media thickness with history of GDM (subgroup analysis)

**Figure 4:**
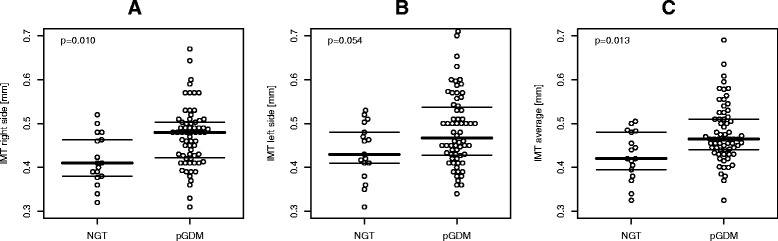
**Bee swarm plot of IMT data in a subgroup of pGDM and controls: right side (A), left side (B), average (C).** Lines indicate first, second (median) and third quartiles.

Baseline parameters associated with IMT (average) were age (β = 0.005, 95%CI 0.002 to 0.008, p = 0.003) and moreover status of GDM during pregnancy (β = 0.046, 95%CI 0.010 to 0.082, p = 0.013, Figure 4), whereas no association was observed between IMT and endothelial biomarkers (sICAM-1: p = 0.098, sVCAM-1: p = 0.581, sE-selectin: p = 0.800) or SI_FSIGT_ (p = 0.176) in univariable analyses. A trend was observed for BMI (β = 0.003, 95%CI 0.00 to 0.006, p = 0.054). A multivariable model revealed that the effects of age and GDM status remained significant after additional adjustment for time after index pregnancy.

## Discussion

In the present study we aimed to assess the relationship between impaired insulin sensitivity and soluble parameters of endothelial dysfunction in females with history of GDM and observed inverse associations between insulin sensitivity estimated from an FSIGT and sICAM-1, sVCAM-1 as well as sE-selectin 3–6 month after delivery. In particular, sICAM-1 and sVCAM-1 were shown to be significantly elevated in pGDMs as compared to females after uncomplicated pregnancy (controls) independent of age, BMI and degree of insulin resistance. As an additional finding our data indicates that circulating biomarkers of endothelial dysfunction were related to parameters of subtle inflammation and tended to be increased in females with diabetes progression during a follow-up period of up to 10 years.

The link between circulating biomarkers of endothelial dysfunction and impaired carbohydrate metabolism or type 2 diabetes has been assessed by previous observational studies: With particular focus on older females (average of 56 years) data from the Nurses’ Health Study indicated that endothelial biomarkers predicted the development of overt diabetes [29]. This observation was also confirmed by another multiethnic cohort of U.S. postmenopausal women [30]. As endothelial biomarkers (e.g. sICAM-1 and sE-selectin) were also related to cardiovascular prognosis in initially health individuals [7] it was hypothesized that dysfunctional endothelium represents a shared precursor of atherosclerosis and CVD (if large arteries are affected) as well as insulin resistance and type 2 diabetes (if endothelial dysfunction addresses capillaries of insulin sensitive tissues) [8,29]. These considerations are also supported by our data as we found an inverse association between insulin sensitivity as well as subtle inflammation and indicators of endothelial dysfunction in our study of relatively young females at a very early stage of cardiometabolic disorders. Our findings are in line with molecular and pathophysiological consequences of insulin resistance in endothelial cells [1,2,6]: While in metabolically healthy individuals insulin acts as a vasoactive hormone, primarily by activating phosphatidylinositol 3-kinase (PI3K) and consequently nitric oxide production (due to Akt mediated phosphorylation of endothelial nitiric oxide synthase), this signaling pathway is notably blunted by insulin resistance leading to impaired endothelium dependent vasodilatation. However, due to compensatory increased insulin release from the β-cells (hyperbolic association between insulin sensitivity and insulin secretion [31]), insulin signaling via the mitogen-activated protein kinase (MAPK) is stimulated, mediating expression of proinflammatory parameters. Hence, the expression of ICAM-1, VCAM-1 or E-selectin on the endothelial surface is over activated with consequently increased soluble levels in insulin resistant individuals, also observed in our study in reproductive aged females early after delivery. In addition, hyperglycemia might affect the Gas6 (growth arrest-specific protein 6)/Axl/Akt signaling pathway in human microvascular endothel cells, as in observational and in vitro studies Gas6 and Gas6/Axl expression was found to be downregulated by increased glucose concentrations [32,33]. This might further impact viability of endothelial cells, angiogenesis and adhesion function and hence might represent a possible target for future therapeutic strategies [33].

In accordance with these pathophysiological considerations particularly sICAM-1 was well explained by glycemic parameters and moreover significantly related to subclinical inflammation and thrombosis in uni- and multivariable analyses (21.2% of the variance of sICAM-1 was explained by the covariables). In contrast, sVCAM-1 was less explained in multivariable analysis (8% of variance) and failed to show any association with incident type 2 diabetes. This is in line with previous observations in mixed populations, as sICAM-1 was shown to predict symptomatic disorders in apparently healthy individuals (as supposed for reproductive aged females), whereas sVCAM-1 is more useful as a risk marker in subjects with established disease [7]. Accordingly, another recent study found significantly increased secretion of ICAM-1 and other markers of endothelial cell dysfunction in human omental tissue of women with obesity or GDM, whereas this was not observed for VCAM-1 expression or secretion [34]. In contrast to sICAM-1 and sVCAM-1 (which are also expressed by other tissues) sE-selectin represents a more endothelial specific parameter, which appeared to be related to subtle inflammation as well as to type 2 diabetes development. However, it has to be mentioned, that in our study the predictive performance for diabetes progression for sE-selectin (which was indicated by univariable analysis) was blunted after adjustment for age and particularly BMI. Therefore, we agree with Chao et al., that from an epidemiological point of view parameters of endothelial dysfunction might contribute only little for diabetes prediction as compared to traditional risk factors including fasting glucose, body composition and clinical risk factors (as was shown by the authors in a large sample of postmenopausal women) [35]. This was also reflected by another recent report, where sE-selectin levels correlated with components of the metabolic syndrome, but however, were not independently related to abnormal glucose regulation early after delivery [36]. We have previously examined several risk factors for diabetes progression by using data of the present study and have identified parameters of disturbed carbohydrate metabolism (e.g. 1 h-post load glucose levels which were also shown to be significantly related to adhesion molecules) [17] but also an index indicating non alcoholic fatty liver disease [20] which might perform superior for risk prediction as compared to the endothelial biomarkers investigated in this study. However, we suggest that the systematic analyses of circulating markers of endothelial dysfunction in longitudinal studies are necessary for thoroughly understanding the development of diabetic and cardiovascular disorders as well as the interrelation between both diseases in women following pregnancy with GDM. Moreover, elevated markers of endothelial dysfunction and oxidative stress might be also elevated in the cord blood of females with GDM [37]. Thus, there is also need for further research concerning the consequences of maternal hyperglycemia particularly in the view of an early onset of endothelial dysfunction and atherosclerosis in the offspring due to the concept of “fetal programming”.

With regard to CVD there is emerging evidence for a markedly increased risk for subjects with history of GDM [38,39], however, data with clearly defined cardio-vascular endpoints in this young aged population are sparsely available. In this study we assessed subclinical atherosclerosis as a secondary objective and in accordance with others [40,41] observed significantly higher IMT values in pGDMs as compared to females after uncomplicated pregnancy, further supporting that history of GDM should be considered as an independent vascular risk factor [41], comparable to the “metabolic syndrome” [40]. IMT measurements were found to be further associated with age, but not with adhesion molecules, measured at the baseline examination. This is somewhat unexpected and might be observed by chance due to the limited number of IMT measures for this secondary objective. However, previous studies reported conflicting results: While one observational study reported significant associations between IMT levels and sICAM-1 as well as sE-selectin 6.5 years following an index pregnancy with GDM [41], this association was not revealed for older females in another study [42]. Although, the results of the letter study indicated a markedly association between sICAM-1 and CVD death in women [42].

Strengths and potential limitations of this report should be addressed: The longitudinal study design as well as repeated measurements of endothelial biomarkers (over a period of five years of observation), as well as the prospective reassessment of glucose tolerance status by OGTTs (for up to 10 years) and the availability of FSIGT data for estimating insulin sensitivity and β-cell function at the baseline visit are the major strength of our study. It might be criticized, that endothelial dysfunction was indirectly assessed by using measurements of circulating biomarkers. Flow-mediated vasodilatation might represent another popular (but also indirect) measurement of endothelial dysfunction. However, we decided to use the more simple approach, due to the large number of repeated measures. In addition, it has to be mentioned, that the evidence of some of our results might be restricted according to the sparse number of subjects with progression to overt diabetes, and hence should be interpreted in a descriptive manner.

## Conclusions

In summary, we found that biomarkers of endothelial dysfunction are related to impaired insulin sensitivity and moreover notably increased in females with recent history of GDM. Thereby sICAM-1 and sE-selectin tended to be elevated in subjects who showed a progression to type 2 diabetes after delivery and hence possibly indicate the later development of overt cardiometabolic disorders in this relatively young aged risk population. Follow-up examinations with clearly defined cardiovascular endpoints as well as randomized trials are necessary to further examine clinical consequences and possible therapeutic approaches. However, our results indicate a need of early risk stratification immediately after delivery. Clinical re-examinations including risk factor modification and patient education might represent an opportunity to prevent disease progression in females with history of gestational hyperglycemia.
